# Mapping super-resolution image quality

**DOI:** 10.1038/s41377-024-01379-4

**Published:** 2024-02-01

**Authors:** Megan A. Steves, Ke Xu

**Affiliations:** Department of Chemistry, University of California, Berkeley, CA 94720 USA

**Keywords:** Super-resolution microscopy, Imaging and sensing

## Abstract

The local quality of super-resolution microscopy images can be assessed and mapped by rolling Fourier ring correlation, even when image quality varies within a single image.

## Graphical abstract

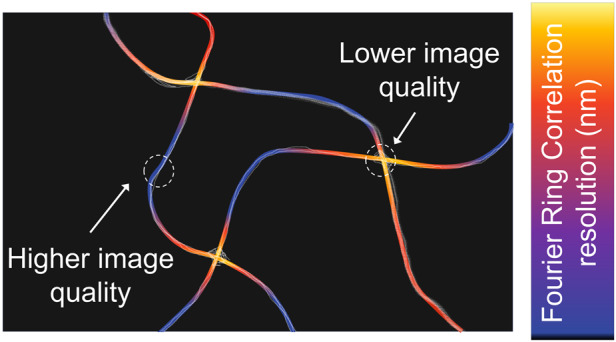

Super-resolution fluorescence microscopy—recognized by the 2014 Nobel Prize—has allowed researchers to visualize biological structures and beyond in exquisite detail. As super-resolution microscopy has become more widely adopted, variations in both experimental techniques and analysis algorithms have proliferated. Consequently, microscopists seeking the best possible resolution for a particular sample could benefit from choosing the most suitable experimental parameters and analysis. Large benchmarking studies offer quantitative measures of image reconstruction quality that can help guide these decisions^1^. However, these benchmarks also demonstrate that no single method performs best in all situations, requiring quantitative assessments of image quality on each dataset. Also, unlike benchmarking studies, methods for determining the quality of experimental images must function using only the data themselves, since ground truth images are not available.

One way to assess the experimental super-resolution image quality is to compare two images of the same sample acquired and analyzed under identical conditions. Differences between the two images likely arise due to errors from experimental noise and other factors, indicating areas where the super-resolution image is less reliable. Simply subtracting the two images is not ideal—intensity fluctuations likely dominate the different images, and small variations in the sample structure may be overshadowed. Fourier ring correlation (FRC), previously introduced for fluorescence microscopy by Nieuwenhuizen et al.^2^, compares images in the frequency domain instead. The Fourier-transformed spatial data from the image pairs are compared through a correlation function. The correlation will be high at frequencies (inverse length) containing meaningful information; at frequencies with unreliable information, the correlation will decay to zero. The frequency at which the correlation drops below a threshold yields the effective resolution of the image. While FRC is commonly used to assess image quality, it gives limited information about how resolution varies across an image, which can be caused by factors such as inhomogeneous labeling density and illumination, optical aberrations including defocusing, and detector defects.

In this issue of LSA, Zhao et al. introduce an approach to address this issue and map out local image quality at high spatial resolution^3^. Their rolling Fourier ring correlation (rFRC) method uses a scanning window to calculate the local FRC (Fig. 1a) and hence maps out the image quality pixel-by-pixel across the image (Fig. 1b). To account for systematic errors, the authors further incorporate an additional module to generate a resolution-scaled error map (RSM)^4^, which identifies artifacts of the reconstruction algorithm by comparing with a diffraction-limited reference image. The authors demonstrate the utility of the combined methodology on both simulated and experimental data. As the method is model-independent, the authors successfully assess super-resolution images acquired with a range of modalities, including 2D and 3D SMLM, SRRF, SIM, and others.

**Fig. 1:**
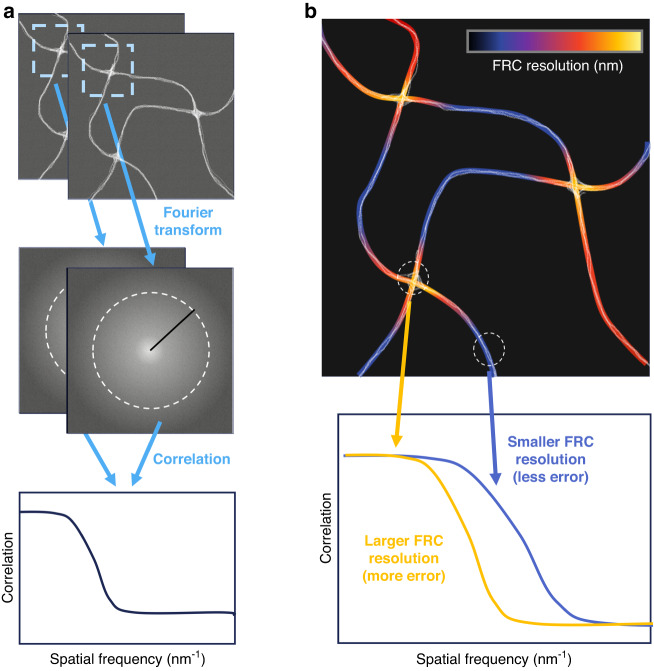
Rolling Fourier ring correlation (rFRC) maps out the local quality in super-resolution images. **a** Starting with two images acquired under identical conditions, a window scans across the image pair so that the selected local super-resolution images are correlated through their Fourier transforms to obtain a local FRC resolution to be assigned to the center of the window. After sliding the window across the entire image, a map **b** of the FRC resolution is obtained, presenting the local image quality

One enabling application of rFRC is the generation of super-resolution images using multiple reconstruction algorithms to achieve the highest resolution across the entire image, depicted in Fig. 2. The various algorithms available for SMLM reconstruction are often best suited for different conditions, such as low or high emitter density. Since emitter density is often heterogeneous across the sample, researchers may need to compromise on an algorithm that is suitable for all the local densities found in their sample. rFRC provides a path to locally determine the best reconstruction method and generate a fused image composed of the best parts of each reconstructed image.

**Fig. 2:**
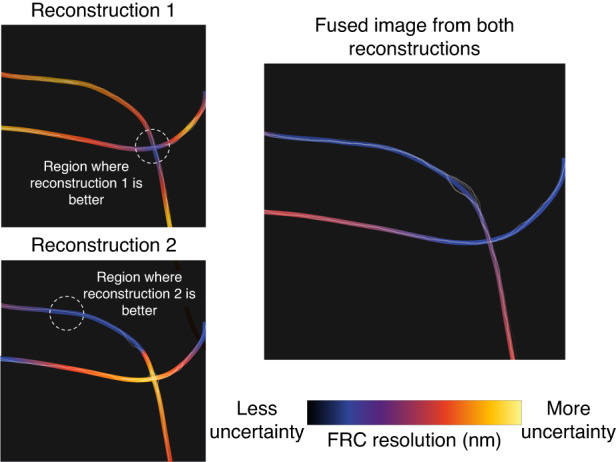
Multiple reconstructions can be combined to improve overall image quality with rFRC. The rFRC maps are used to guide the fusion of the highest quality regions from images using two different reconstruction algorithms, generating a higher quality image

The results reported by Zhao et al. reflect a contribution towards reproducible and quantitative super-resolution microscopy. With implementations for ImageJ, MATLAB, and Python, rFRC joins other open-source toolboxes such as NanoJ-SQUIRREL^4^, HAWKMAN^5^, and SIMCheck^6^ for assessing super-resolution image quality, alongside the numerous available algorithms for reconstructing super-resolution images from raw data. Ultimately, the resulting quality of super-resolution images is a product of both experimental parameters and the image processing steps used to create them. Quantitative analysis of image quality can generate more confidence in the results of super-resolution studies, avoid overinterpretation of images, and suggest new paths towards even higher resolution.

From a different perspective, by mapping out local image quality at the super-resolution level, rFRC also echoes recent efforts to extend super-resolution microscopy to new dimensions^7^. The possibility to encode new functional information^8^ into such high-dimensional super-resolution maps, or, in the opposite direction, the generalization of rFRC to map out the local quality of or variations in other multidimensional super-resolution data, afford attractive prospects.

## References

[1] Sage D (2019). Super-resolution fight club: assessment of 2D and 3D single-molecule localization microscopy software. Nat. Methods.

[2] Nieuwenhuizen RPJ (2013). Measuring image resolution in optical nanoscopy. Nat. Methods.

[3] Zhao WS (2023). Quantitatively mapping local quality of super-resolution microscopy by rolling Fourier ring correlation. Light Sci. Appl..

[4] Culley S (2018). Quantitative mapping and minimization of super-resolution optical imaging artifacts. Nat. Methods.

[5] Marsh RJ (2021). Sub-diffraction error mapping for localisation microscopy images. Nat. Commun..

[6] Ball G (2015). SIMcheck: a toolbox for successful super-resolution structured illumination microscopy. Sci. Rep..

[7] Xiang LM, Chen K, Xu K (2021). Single molecules are your quanta: a bottom-up approach toward multidimensional super-resolution microscopy. ACS Nano.

[8] Yan R, Wang BW, Xu K (2019). *Functional* super-resolution microscopy of the cell. Curr. Opin. Chem. Biol..

